# High expression of *AFAP1-AS1* is associated with poor survival and short-term recurrence in pancreatic ductal adenocarcinoma

**DOI:** 10.1186/s12967-015-0490-4

**Published:** 2015-04-30

**Authors:** Yibiao Ye, Jie Chen, Yu Zhou, Zhiqiang Fu, Quanbo Zhou, YingXue Wang, Wenchao Gao, ShangYou Zheng, Xiaohui Zhao, Tao Chen, Rufu Chen

**Affiliations:** Department of Hepatobiliary Surgery, Sun Yat-sen Memorial Hospital, Sun Yat-sen University, Guangzhou, China; Department of Pancreaticobiliary Surgery, Hepatobiliary Surgery, Sun Yat-sen Memorial Hospital, Sun Yat-sen University, Guangzhou, China; Department of Medical Oncology, Sun Yat-sen Memorial Hospital, Sun Yat-sen University, Guangzhou, China; Department of General Surgery, The Second Affiliated Hospital of Sun Yat-sen University, Sun Yat-sen University, 107 Yan-Jiang Xi Road, Guangzhou, 510120 China; Present Address: Department of General Surgery, Guangdong General Hospital, Guangzhou, China

**Keywords:** Pancreatic ductal adenocarcinoma, AFAP1-AS1, Long noncoding RNAs

## Abstract

**Background:**

Pancreatic ductal adenocarcinoma (PDAC) is still a lethal malignancy. Long noncoding RNAs (lncRNAs) have been shown to play a critical role in cancer development and progression. Here we identified overexpression of the lncRNA *AFAP1-AS1* in PDAC patients and evaluated its prognostic and functional relevance.

**Methods:**

The global lncRNA expression profile in PDAC was measured by lncRNA microarray. Expression of *AFAP1-AS1* was evaluated by reverse-transcriptase quantitative polymerase chain reaction (RT-qPCR) in 90 PDAC tissue samples and adjacent normal tissues. The impact of *AFAP1-AS1* expression on cell proliferation, migration, and invasion were evaluated *in vitro* using knockdown and ectopic expression strategies.

**Results:**

Microarray analysis revealed that up-regulation of *AFAP1-AS1* expression in PDAC tissues compared with normal adjacent tissues, which was confirmed by RT-qPCR in 69/90 cases (76.7%). Its overexpression was associated with lymph node metastasis, perineural invasion, and poor survival. When using *AFAP1-AS1* as a prognostic marker, the areas under ROC curves were 0.8669 and 0.9370 for predicting tumor progression within 6 months and 1 year, respectively. *In vitro* functional experiments involving knockdown of *AFAP1-AS1* resulted in attenuated PDAC cell proliferation, migration, and invasion. Ectopic expression of *AFAP1-AS1* promoted cell proliferation, migration, and invasion.

**Conclusions:**

*AFAP1-AS1* is a potential novel prognostic marker to predict the clinical outcome of PDAC patients after surgery and may be a rational target for therapy.

**Electronic supplementary material:**

The online version of this article (doi:10.1186/s12967-015-0490-4) contains supplementary material, which is available to authorized users.

## Background

Pancreatic ductal adenocarcinoma (PDAC) remains one of the most aggressive human cancers [1]. Despite substantial efforts, PDAC is associated with a short survival that has been declining steadily since the early 1990s [2]. PDAC is characterized by a highly malignant phenotype that is associated with early metastasis and resistance to chemotherapy and radiation therapy [3]. There is an urgent need for further understanding of the mechanism of PDAC development and new innovative therapeutic approaches. Identifying the underlying molecular mechanisms of invasion and metastasis in PDAC will be essential for the identification of effective drug targets.

In recent years, it has become increasingly apparent that the noncoding portion of the genome is of crucial functional importance in both normal physiology and diseases [4]. Long noncoding RNAs (lncRNAs), which are defined as those longer than ~200 nucleotides but lacking protein coding capacity [3], have recently been shown to play a key role in regulating vital cellular functions including cancer progression [5]. To date, thousands of lncRNAs have been discovered through chromatin signature analysis and large-scale sequencing, and functional studies have shown that many of them exhibit diverse biological functions and have clinical significance [6]. Importantly, many lncRNAs have been identified as being cancer-specific [5,7]. For example, aberrant expression of lncRNA *HOTAIR* was associated with various cancers such as breast, hepatocellular, gastric, colorectal, and pancreatic, and its expression was associated with survival and prognosis of cancer patients [8]. *MALAT1* was discovered as a prognostic marker for lung cancer metastasis but also been linked to several other human malignancies [9]. Other examples include HULC in hepatocellular carcinoma [10] and PCGEM1 in prostate cancer [11,12]. In pancreatic, a number of lncRNAs were found to exhibit pro-oncogenic or tumor-suppressive activities, such as ENST00000480739 [13], *LOC285194* [13], *HULC* [14], *HOTAIR* [15], and *MALAT1* [16], suggesting an important of lncRNAs in progression of pancreatic cancer. Therefore, identification of lncRNAs involved in PDAC progression might help yield novel prognostic biomarkers or therapeutic targets.

In this study, we observed that a lncRNA, *AFAP1-AS1*, was substantially overexpressed in PDAC tissues. Knockdown of *AFAP1-AS1* could inhibit cell proliferation, migration, and invasion of PDAC cells. Moreover, *AFAP1-AS1* expression correlated with lymph node metastasis, perineural invasion, and poor survival in PDAC patients. Our results suggest that *AFAP1-SA1* may represent a novel indicator of poor prognosis and a potential therapeutic target in PDAC.

## Methods

### Cell culture

The human pancreatic cancer cell lines Panc1 (CRL-1469™), MIAPaCa-2(CRL-1420™), Capan2(HTB-80™), SW1990(CRL-2172™), and BXPC-3 (CRL-1687™), and the human pancreatic ductal epithelial cells line HPDE6 were purchased from the American Type Culture Collection and grown in complete growth medium as recommended by the supplier with 10% fetal bovine serum (FBS) and 1% penicillin/streptomycin. All cells were cultured in a humidified 5% CO_2_ incubator at 37°C.

### RNA Isolation, quantitative real-time reverse-transcription polymerase chain reaction (PCR), and microarrays

Quantitative real-time PCR (RT-qPCR) was performed for *AFAP1-AS1* and the epithelial–mesenchymal transition (EMT) markers E-cadherin, N-cadherin, Vimentin, Snai1, and Slug. β-Actin was used as an internal control. RNA was extracted from frozen pancreatic cancer tissues and their corresponding non-neoplastic tissues and pancreatic cell lines using TRIzol reagent (Invitrogen, Carlsbad, CA, USA). The total RNA was then converted to cDNA by reverse-transcription using oligodT primers and SuperScript II reverse transcriptase (Invitrogen). For real-time quantitative PCR, three replicates of each sample were amplified and analyzed using a Roche Light-Cycler (Roche, Basel, Switzerland). The 20 μl reaction mixtures contained SYBR Green reaction mix (Qiagen Sciences) and 0.5 mM of primer. Relative gene expression was determined using the comparative delta-delta CT method (2-∆∆Ct). The primer sequences for PCR were provided in the supplementary materials (Additional file 1).

Transcriptomic analysis was performed using Arraystar human lncRNA microarrays, V3 (Agilent, USA), which target 27958 Entrez protein-coding genes and 7419 lncRNAs. Total RNA was extracted and mRNA was purified using the mRNA-ONLYTM Eukaryotic mRNA Isolation Kit (Epicentre). Total RNA was fragmented, labeled (One-Color, Cy3, Agilent), purified, and hybridized with probes in Hybridization Chamber gasket slides (Agilent). The slides were then washed and scanned with an Agilent Microarray Scanner. The raw data were extracted with Agilent Feature Extraction software (Agilent Technology). This software uses the robust multi-array average algorithm to adjust the background signals. Normalized data were obtained after performing the quantile method of intra-microarray normalization and the median method of baseline transformation between the microarrays. Differentially expressed genes with a raw expression level of >400 in more than 4 out of the 12 samples used for profiling were extracted and ordered by p-value. Genes with the highest top 10 p-values were selected for validation. The microarray platform and data were submitted to the Gene Expression Omnibus public database at the National Center for Biotechnology Information (accession number: GSE61166, http://www.ncbi.nlm.nih.gov/geo/query/acc.cgi?acc=GSE61166).

### Immunoblotting

Immunoblotting was performed as described previously [17]. Briefly, cells were washed in phosphate-buffered saline (PBS) and lysed with RIPA buffer (Invitrogen, Carlsbad, CA) plus protease inhibitor cocktail (Roche, Mannheim, Germany). For equal protein loading, a bicinchoninic acid protein assay kit (Pierce) was used to calculate protein concentration in each sample. Equivalent amounts of proteins were subjected to sodium dodecyl sulfate polyacrylamide gel electrophoresis, transferred to a polyvinylidene fluoride membrane, blocked in 5% fat-free milk for 2 hours at room temperature, and detected with appropriate primary antibodies. The following antibodies were used for analysis: anti-E-cadherin (1:1000; BD Biosciences, CA, USA), anti-N-cadherin (1:1000; BD Biosciences), anti-Vimentin (1:1000; BD Biosciences), anti-slug (1:1000; Abcam), anti-snail (1:1000, Abcam), and anti-β-actin (Sigma). β-Actin was used for loading controls. Horseradish peroxidase-conjugated secondary antibodies (Cell Signaling Technology), and an ECL chemiluminescence kit (Pierce) were used to detect bound antibody.

### *AFAP1-AS1* knockdown

#### Vector construction and virus infection

For lentivirus-mediated suppression of human *AFAP1-AS1*, the following shRNA and scrambled control shRNA were inserted into the pMKO.1-puro vector: #1, forward, 5′-CCGGAGCGGTCTCAGCCGAATGACTCTCGAGAGTCATTCGGCTGAGACCGCTTTTTTG-3′ and reverse, 5′-AATTCAAAAAAGCGGTCTCAGCCGAATGACTCTCGAGAGTCATTCGGCTGAGACCGCT-3′; #2, forward, 5′-CCGGAACACCAATCCCAAGAGGTGACTCGAGTCACCTCTTGGGATTGGTGTTTTTTTG-3′ and reverse, 5′-AATTCAAAAAAACACCAATCCCAAGAGGTGACTCGAGTCACCTCTTGGGATTGGTGTT-3′; scrambled control shRNA, forward 5′-CCGGTTTCTCCGAACGTGTCACGTCTCGAGACGTGACACGTTCGGAGAATTTTTG-3′and reverse, 5′-AATTCAAAAAGTTCTCCGAACGTGTCACGTCTCGAGACGTGACACGTTCGGAGAA-3′. For ectopic expression of *AFAP1-AS1*, The full-length *AFAP1-AS1* cDNA was generated by reverse transcriptase-polymerase chain reaction (RT-PCR) using total RNA from SW1990 pancreatic cancer cell line. The *AFAP1-AS1* cDNA with the wild-type sequence was inserted into the EcoRI site of the pcDNA3.1(+) expression vector (Invitrogen) to obtain the *AFAP1-AS1*wt/pcDNA3.1(+) construct. Lentivirus packaging, cell infection and selection of puromycin-resistant cells was performed as previously described [17].

### Cell growth and cell cycle assays

Cell proliferation analysis 3-(4,5-dimethylthiazol-2-yl)-2,5-diphenyltetrazolium bromide (MTT) substrate (Sigma-Aldrich) was used to assay cell proliferation according to the manufacturer’s instructions. Briefly, a total of 3 × 10^3^ cells were seeded into 96-well dishes and allowed to adhere overnight. The growth curves of cells, covering a total of 3 days of culturing, were determined through measuring absorbance at 570 nm. After transfection, 5 × 10^4^ SW1990, MIAPaCa-2 cells, or PANC-1 cells were collected and washed three times with PBS. After RNase digestion and PI dyeing, the cells were subjected to FACS analysis.

### Migration and invasion assays

The cell migration assay was performed using BD Transwell chambers. Cell invasion assays were performed with chambers uniformly covered with Matrigel (BD Biosciences) diluted with Dulbecco’s modified Eagle’s medium (DMEM) to a certain percentage and incubated at 37°C for 30 minutes. Cells (1 × 10^6^) were suspended in 200 μl serum-free DMEM medium and seeded into the upper chamber of each insert. Then, 600 μl of DMEM containing 10% FBS was added to a 24-well plate. After incubation at 37°C for 12 h, the cells that migrated were fixed and stained for 20 min in a dye solution containing 0.4% crystal violet and 20% methanol. The cells in the upper layer of the membrane were removed and the cells in the lower layer were washed off with 33% acetic acid (500 μl per chamber). The migrated cells was quantified by measuring the absorbance of eluent at 570 nm. The relative migration fold change of the experimental group was calculated by normalizing to that of the control group.

### Patient samples

All samples were obtained from patients when undergoing resection of the pancreas at Sun Yat-Sen Memorial Hospital between 2009 and 2014. Informed consent was obtained from the patients before sample collection. All patients had a clear histological diagnosis. Patients’ specimens and the related clinicopathological data, including complete follow-up, were obtained from the Institute of Pathology and from the Department of Pancreaticobiliary, Sun Yat-Sen Memorial Hospital. All patients in this study met the following criteria: 1) PDAC diagnosis was verified by pathological examination; 2) paraffin-embedded tissues were well stored and qualified for serial section; 3) the corresponding tumor tissues and the paired non-tumor tissues were stored in liquid nitrogen immediately following surgical removal; 4) no anticancer treatments given before biopsy collection; and 5) availability of exhaustive clinicopathologic and follow-up data.

### Tumorigenicity assays in nude mice

All experiments involving animals were conducted according to the institutional guidelines of Guangdong Province and were approved by the institutional guidelines of Guangdong Province and by the Use Committee for Animal Care. BALB/c nude mice (5 weeks old) were randomly separated into the shControl group or the sh*AFAP1-AS1* group (5 mice per group). SW1990 cells (3 × 10^6^ cells/mouse) stably transfected with sh*AFAP1-AS1* or control shRNA were injected subcutaneously into the right axilla of each mouse. Tumor volume was calculated using the following formula: volume = (L × W^2^)/2, where L and W are the longest and shortest diameters, respectively. The mice were sacrificed two weeks after injection.

### Statistical analysis

Statistical analyses were performed using SPSS Statistics 17.0 (SPSS lnc®). The chi-square test (*X*^2^ test), Fisher’s exact test for nonparametric variables, and Student’s *t* test for parametric variables (two-tailed) were used. Differences in patient survival were assessed using the Kaplan–Meier method and analyzed using the log-rank test in univariate analysis. All tests were two tailed, and results with *P* = 0.05 were considered statistically significant.

## Results

### *AFAP1-AS1* is aberrantly overexpressed in human PDAC cell lines and cancerous tissues

**Figure 1:**
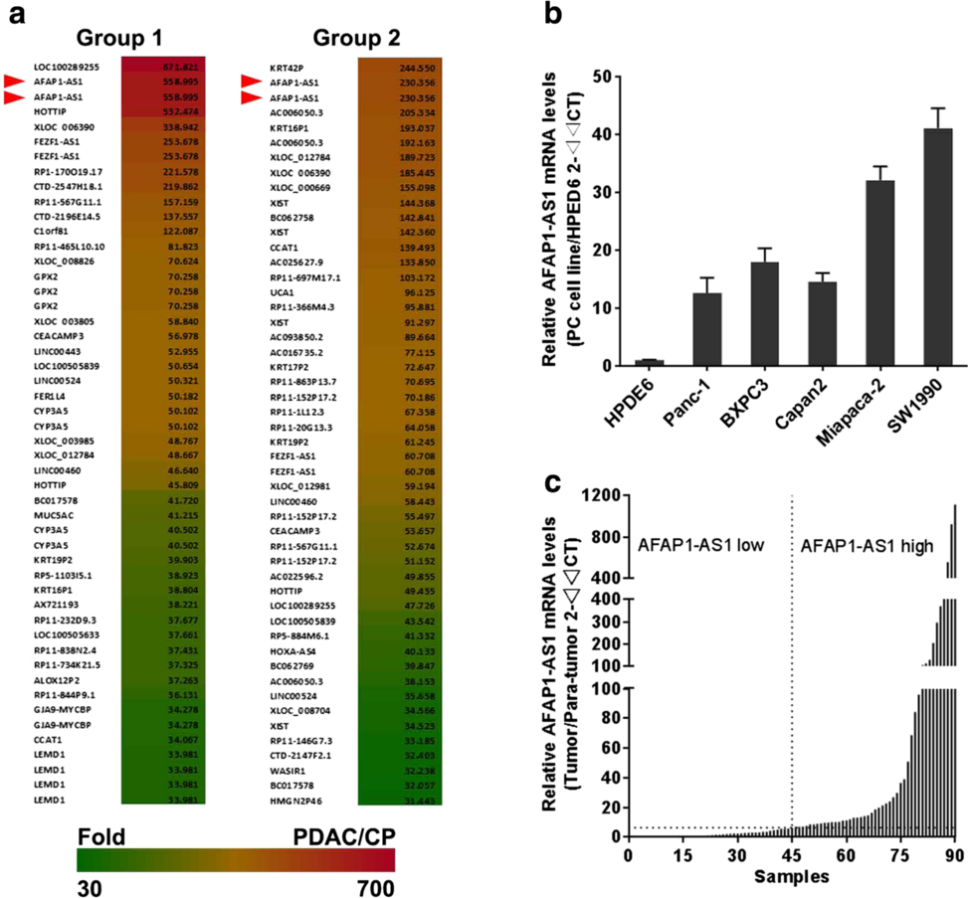
*AFAP1-AS1* expression in clinical pancreatic ductal adenocarcinoma specimens and cancer cell lines. **(a)** Heat map representing the top 50 up-regulated long noncoding RNAs in tissue from eight cases of pancreatic ductal adenocarcinoma (PDAC) compared with tissue from four cases of chronic pancreatitis (CP). Group 1 indicates the most up-regulated lncRNAs in PDAC tissues from patients with diabetes, and group 2 indicated the most up-regulated lncRNAs in PDAC tissues from patients without diabetes. **(b)** The *AFAP1-AS1* expression levels in PDAC cell lines were determined via RT-qPCR, and β-actin was used as internal control. Data are represented as the mean ± s.d. from three independent experiments. ***: *P* < 0.01, Student’s *t*-test. **(c)** *AFAP1-AS1* expression levels in 90 paired PDAC tissues and their adjacent noncancerous tissues were examined via RT-qPCR. The relative *AFAP1-AS1* mRNA level was normalized to β-actin.

As a first attempt to identify differentially expressed long noncoding RNAs (lncRNAs) in two subtypes of PDAC tissues (PDAC patients with diabetes versus PDAC patients without diabetes), we conducted microarray analysis utilizing a microarray targeting 7419 LncRNAs using eight cases of PDAC tissues and four cases of chronic pancreatitis tissues (CP) (accession number: GSE61166). All of the differentially expressed (> or <2 fold change) lncRNAs were listed in Additional file 2. We noticed that the long noncoding RNA *AFAP1-AS1* is one of the most up-regulated lncRNAs in both subtypes of PDAC tissues (Figure 1a), suggesting a potentially important role for *AFAP1-AS1* in PDAC development. Therefore, we next examined the expression of *AFAP1-AS1* in multiple PDAC cell lines. We observed that the expression level of *AFAP1-AS1* in each PDAC cell line was dramatically up-regulated compared with the HPDE6 cell line (human pancreatic ductal epithelium cell; Figure 1b). Finally, we sought to identify whether *AFAP1-AS1* was up-regulated in PDAC cell lines and in a large sample size of PDAC tissues. As shown in Figure 1c, we discovered that *AFAP1-AS1* was widely upregulated in PDAC tissues compared their paired adjacent non-tumor tissues.

### High *AFAP1-AS1* expression predicts poor prognosis in PDAC patients with surgical resection

**Figure 2:**
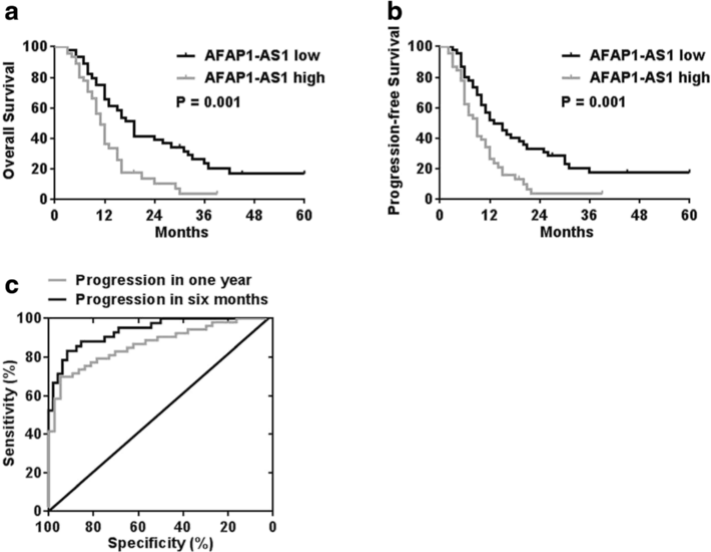
High *AFAP1-AS1* expression predicts poor prognosis in PDAC patients with surgical resection. **a)** and **b)** Patients were equally divided into two groups based on *AFAP1-AS1* mRNA levels. The log-rank test (2-sided) was used to compare differences between groups. The Kaplan–Meier curves show analyses of overall survival and progression-free survival. **(c)** ROC curve analysis was conducted to investigate the value of *AFAP1-AS1* in predicting tumor progression (recurrence and/or metastasis) within 6 months or 1 year after surgical resection.

We next asked whether the expression of *AFAP1-AS1* correlated with the clinical outcome in patients with PDAC. As shown in Figure 1c, 90 cases of PDAC patients received surgical resection were divided into two groups based on *APAF1-AS1* expression with 45 patients in each group. Log-rank analysis indicated that the overall survival and progression-free survival was significantly worse in patients with higher *AFAP1-AS1* expression in their tumor tissues (Figure 2a, b). Statistical analysis also revealed that *AFAP1-AS1* overexpression correlated with lymph node metastasis and perineural invasion (Table 1). No statistical correlation with gender, age, tumor stage and tumor grade was observed. To further determine whether and how *AFAP1-AS1* can serve as a biomarker to predict tumor progression (local recurrence and/or metastasis) after surgery, we constructed a ROC (receiver operating characteristic) curve analysis (Figure 2c). For predicting progression within 1 year, the area under the ROC curve was 0.8669 (p < 0.0001) with an optimal cutoff point of 8.797 (tumor/para-tumor; sensitivity = 69.81%, specificity = 94.59%) and for predicting progression within in 6 months, the area under the ROC curve was 0.9370 (p < 0.0001) with an optimal cutoff point of 8.797 (tumor/para-tumor; sensitivity = 83.33%, specificity = 91.67%). These findings suggest that *AFAP1-AS1* has potential diagnostic value in predicting early recurrence of PDAC.

**Table 1:** **Correlation of** ***AFAP1-AS1*** **expression and clinicopathological characteristics**

**Factors**		***AFAP1-AS1*** **expression**		P value*
		**Higher (n = 45)**	**Lower (n = 45)**	
Age	<60	25	20	0.292
	≥60	20	25	
Sex	Male	29	28	0.827
	Female	16	17	
Differentiation	Well	15	18	0.598
	Moderate	17	18	
	Poor	13	9	
UICC stage	pI	13	11	0.634
	pII	32	34	
T stage	T1	6	9	0.355
	T2	18	13	
	T3	21	13	
N stage	N0	10	27	0.001
	N1	35	18	
Perineural invasion	Negative	18	29	0.020
	Positive	27	16	

*Pearson Chi-Square test.

### Inhibition of *AFAP1-AS1* in PDAC cells leads to reduced proliferation

**Figure 3:**
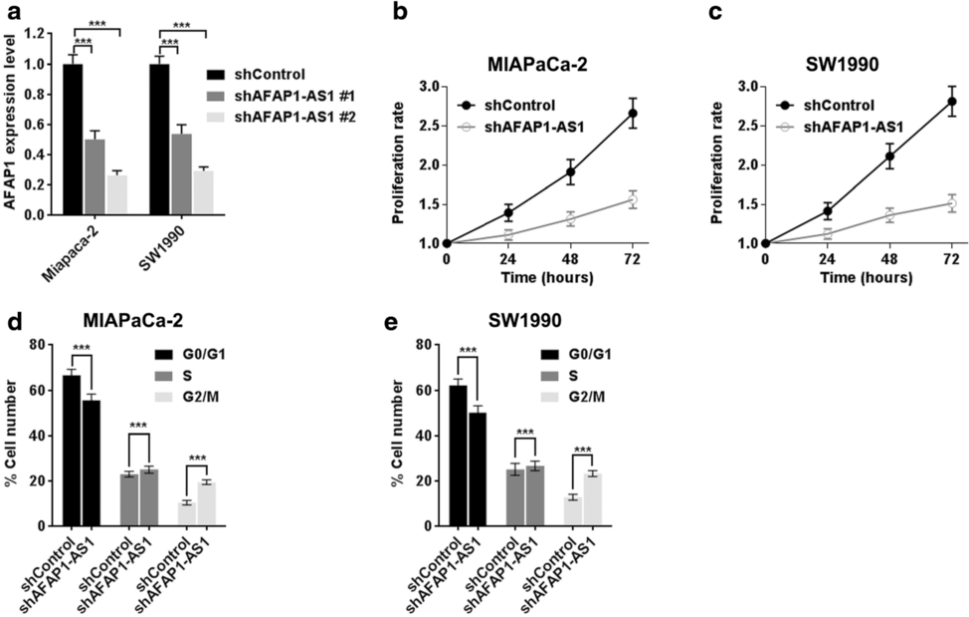
*AFAP1-AS1* knockdown attenuates PDAC cell proliferation. **(a)** *AFAP1-AS1* expression level was confirmed by RT-qPCR. **(b**-**c)** The effect of *AFAP1-AS1* knockdown on cancer cell line proliferation was determined by MTT assays. **(d**-**e)** The effect of *AFAP1-AS1* knockdown on the cell cycle was measured by flow cytometry. Data are represented as the mean ± s.d. from three independent experiments; shControl denotes shRNA having no homology to any known mammalian genes as a negative control. ***: *P* < 0.01, Student’s *t*-test.

To further examine whether *AFAP1-AS1* has a causal role in PDAC progression, *in vitro* functional studies were conducted. We knocked down *AFAP1-AS1* expression in MIAPaca-2 and SW1990 cells via stable transfection, and the efficiency of knockdown of the two shRNAs was evaluated (Figure 3a), the most effective shRNA #2 was chose for the following study. *AFAP1-AS1* depletion resulted in decreased tumor cell proliferation both in PDAC cell line MIAPaca-2 and SW1990, as determined by MTT assay (Figure 3b, c). We also performed cell cycle assays after shRNA transfection using flow cytometry (Figure 3d, e). Results showed that suppression of *AFAP1-AS1* significantly induced G2/M phase arrest. Taken together, these findings suggest that the *AFAP1-AS1* modulates cell proliferation partly through regulating cell cycle.

### *AFAP1-AS1* regulates cell migration and invasion

**Figure 4:**
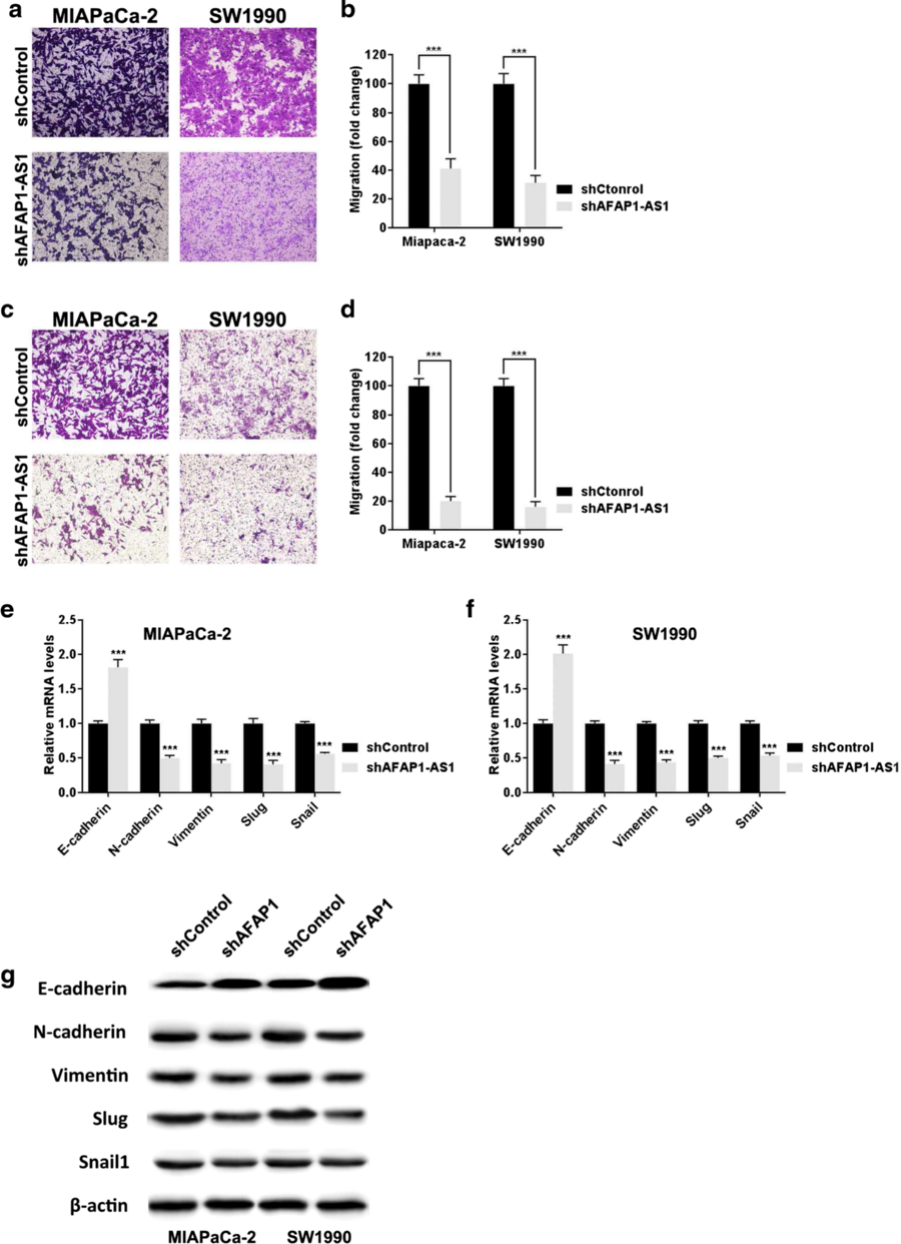
*AFAP1-AS1* knockdown inhibits migration and invasion of PDAC cells. **(a)** Representative images of transwell assay after *AFAP1-AS1* knockdown in PDAC cell line MIAPaCa-2 and SW1990. **(b)** Analysis of OD (570 nm) absorbance of crystal violet-stained cells in transwell assay. **(c)** Representative images of Matrigel invasion assay after *AFAP1-AS1* knockdown in PDAC cell line MIAPaCa-2 and SW1990. **(d)** Analysis of OD (570 nm) absorbance of crystal violet-stained cells in Matrigel invasion assay. **(e**-**f)** Relative mRNA expression levels of EMT-related genes (normalized to β-actin) in PDAC cells after *AFAP1-AS1* knockdown were determined by RT-qPCR. **(g)** Western blot analysis of E-cadherin, N-cadherin, and Vimentin. Data are represented as the mean ± s.d. from three independent experiments, shControl denotes shRNA having no homology to any known mammalian genes as a negative control. ***: *P* < 0.01, Student’s *t*-test.

Enhanced cell migration and invasion abilities are key features associated with cancer metastasis. We therefore examined whether *AFAP1-AS1* knockdown affects these functions in PDAC cells. As shown in Figure 4a and b, *AFAP1-AS1* knockdown significantly decreased cell motility. Similarly, a matrigel invasion assay showed that *AFAP1-AS1* knockdown significantly inhibited invasiveness in MIAPaca-2 and SW1990 cells (Figure 4c, d). Since epithelial–mesenchymal transition (EMT) is closely related with the cell motility and invasiveness, we next examined whether the knockdown of *AFAP1-AS1* affects the expression of EMT-related genes. Both PCR (Figure 4e and f) and Western blot analyses (Figure 4g) showed that suppression of *AFAP1-AS1* in PDAC cells was associated with upregulation of epithelial marker E-cadherin and downregulation of mesenchymal markers.

### Ectopic expression of *AFAP1-AS1* promoted proliferation, migration, and invasion of pancreatic cancer cell

**Figure 5:**
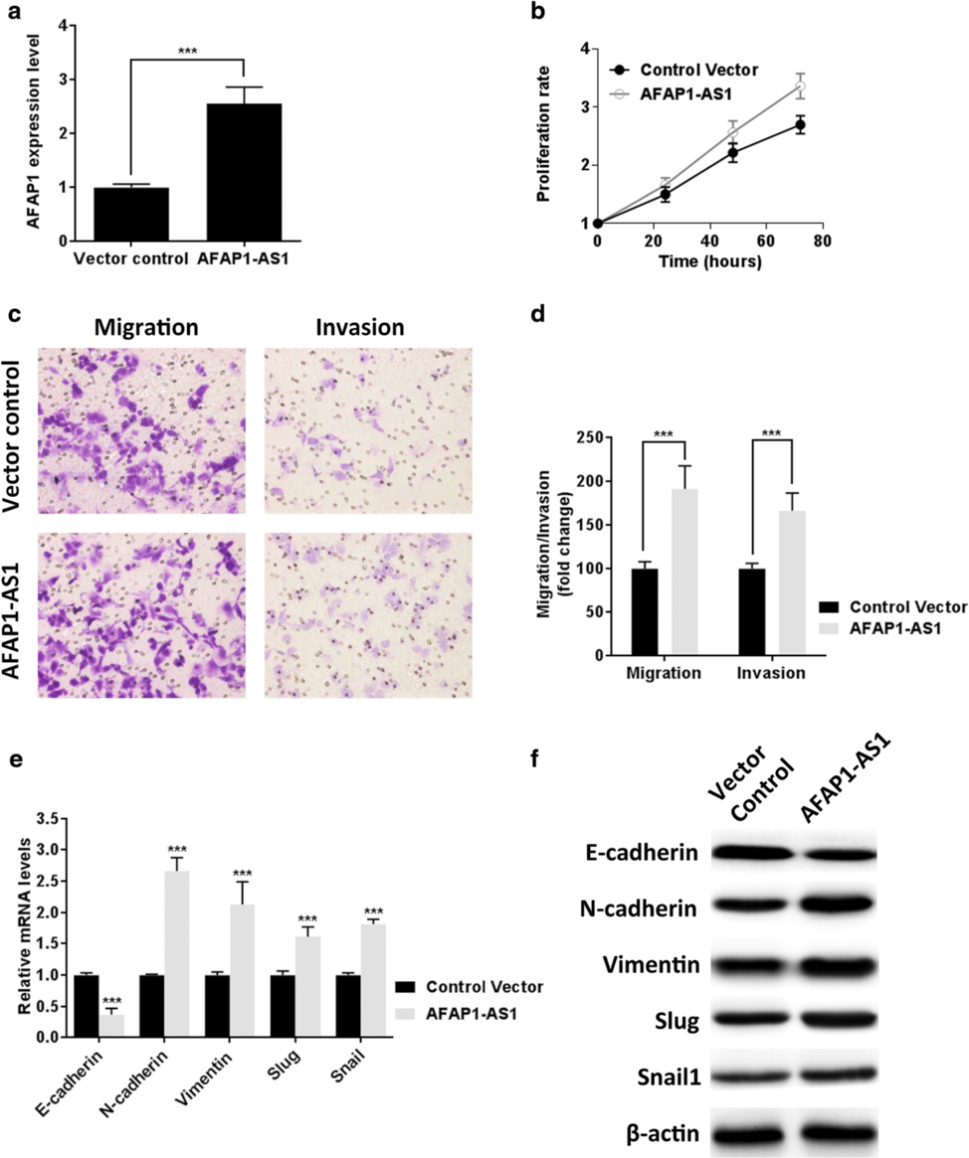
Ectopic expression of *AFAP1-AS1* promoted proliferation, migration, and invasion of pancreatic cancer cell. **(a)** *AFAP1-AS1* expression level was confirmed by qPCR 48 hours after transfection. **(b)** The effect of *AFAP1-AS1* overexpression on PANC-1 cell proliferation was determined by MTT assays. **(c)** Representative images of transwell assay after *AFAP1-AS1* ectopic expression n in PANC-1 cells. **(d)** Analysis of OD (570 nm) absorbance of crystal violet-stained cells in migration assay and invasion assay. **(e)** Relative mRNA expression levels of EMT-related genes (normalized to β-actin) in PDAC cells after *AFAP1-AS1* knockdown were determined by RT-qPCR. **(f)** Western blot analysis of E-cadherin, N-cadherin, Vimentin, Slug, and Snial1. Data are represented as the mean ± s.d. from three independent experiments. Vector control denotes cells transfected with empty vector. ***: *P* < 0.01, Student’s *t*-test.

To further evaluate the oncogenic role of *AFAP1-AS1* in PDAC, ectopic expression of *AFAP1-AS1* was carried out in PANC-1 cells which have the lowest *AFAP1-AS1* level among the five pancreatic cancer cells. Overexpression of *AFAP1-AS1* was confirmed by qRT-PCR (Figure 5a). As expected, *AFAP1-AS1* overexpression resulted in increased tumor cell proliferation (Figure 5b). In addition, migration assay and invasion assay were also performed (Figure 5c), and PANC-1 cells showed significant enhanced migration and invasion ability after *AFAP1-AS1* overexpression (Figure 5d). Both PCR (Figure 5e) and Western blot analyses (Figure 5f) demonstrated that introduction of *AFAP1-AS1* in PANC-1 cells was associated with down of epithelial marker E-cadherin and upregulation of mesenchymal markers.

### Inhibition of *AFAP1-AS1* impaired pancreatic cancer cell tumorigenicity *in vivo*

**Figure 6:**
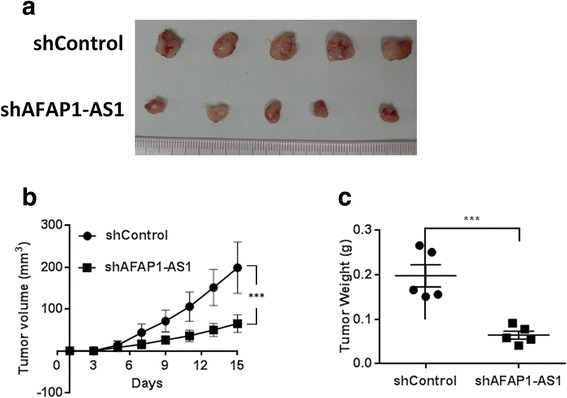
Inhibition of *AFAP1-AS1* impaired pancreatic cancer cell tumorigenicity *in vivo*. **(a) (b)** Nude mice were subcutaneously injected into the right axilla with 3 × 10^6^ cells infected with shControl lentiviral vector (containing scrambled control shRNA) or sh*AFAP1-AS1* lentiviral vector (containing shRNA targeting *AFAP1-AS1*). Tumor growth was then monitored using calipers, and the mice were killed two weeks after injection. **(c)** The tumors were weighed, and compared between the groups. ***: P < 0.01, Student’s *t*-test.

To evaluate the effect of *AFAP1-AS1* on the efficiency of xenograft formation of pancreatic cancer cells, we analyzed the *in vivo* tumorigenicity of SW1990 cells in nude mice following the shRNA-mediated knockdown of *AFAP1-AS1*. As expected, both tumor volume (Figure 6a, b) and tumor weight (Figure 6c) were significant decreased with pancreatic cancer cells when *AFAP1-AS1* expression was inhibited.

## Discussion

Although thousands of lncRNAs have recently been identified, investigation of their respective roles in modulating gene expression is relatively incomplete. Functional studies have indicated that some lncRNAs are involved in human cancer tumorigenesis, progression, and adjuvant therapy resistance, acting as oncogenes or tumor suppressors [4,18]. In the current study, by using high-throughput microarrays, we found that *AFAP1-AS1* is markedly upregulated in PDAC cell lines and in primary material, and its overexpression correlates with lymph node metastasis, perineural invasion, and poor prognosis of PDAC patients. These observations suggest pro-oncogenic activity of *AFAP1-AS1*, a notion that is further supported by our functional studies showing that *AFAP1-AS1* knockdown attenuates PDAC cell proliferation, migration, and invasion.

Lymph node metastasis and perineural invasion are the strongest indicators of short overall survival in PDAC patients. Efforts have recently been made to identify molecular predictive factors in pancreatic cancer patients [19-21]. In the current study, we observed that *AFAP1-AS1* overexpression is associated with lymph node metastasis, perineural invasion, and overall survival after surgical treatment, raising the possibility that this lncRNA may provide a means of identifying high-risk patients for more intensive therapy. Importantly, ROC curve analysis revealed that *AFAP1-AS1* has great potential in predicting tumor progression after surgery. In the data set of our present study, we observed that an over 8-fold increase in *AFAP1-AS1* expression in PDAC tissues compared with adjacent non-tumor tissues was associated with an extremely high risk of short-term recurrence. Whether *AFAP1-AS1* alone, or in combination with other markers, could predict PDAC short-term recurrence required further research through well-designed studies with larger sample size.

In this study, the clinical value of *AFAP1-AS1* in PDAC was supported by functional analysis, which showed that *AFAP1-AS1* suppression diminished migration, invasion, and expression of EMT-related genes in PDAC cells, and *AFAP1-AS1* ectopic expression promoted these malignant behaviors vice versa. This long noncoding RNA was first reported by Wu et al. [22], who showed that *AFAP1-AS1* is overexpressed in primary esophageal adenocarcinoma tissues and regulates esophageal adenocarcinoma cell proliferation, migration, and invasion. Consistent with the latter report, the findings of our study support a similar oncogenic role for *AFAP1-AS1* in PDAC. Importantly, our data showed that *AFAP1-AS1* was one of the most intensely and frequently overexpressed lncRNA in PDAC, further highlighting this transcript to be of significant biological interest in the study of pancreatic cancer pathogenesis.

The *AFAP1-AS1* is derived from the antisense strand of the *AFAP1* (Actin Filament Associated Protein) gene, the sense strand of which encodes the protein AFAP1. The function of the *AFAP1* gene in oncogenesis has been investigated in both breast and prostate cancer. It has been reported that *AFAP1* regulates Src activity and promotes the formation of actin stress fibers and focal adhesions in breast cancer cells [23]. The loss of *AFAP1* in prostate cancer cells reduced cell proliferation and tumorigenesis in nude mice [24]. Usually, the antisense RNA regulates expression of its cognate sense gene, but Wu et al. demonstrated that *AFAP1-AS1* had limited effect on *AFAP1* expression, instead functioning in an almost *AFAP1*-independent manner [22]. Therefore, it will be of great interest in future to investigate whether *AFAP1-AS1* is involved in regulating *AFAP1*. Whether overexpression of *AFAP1-AS1* is induced by hypomethylation or other epigenetic mechanism also deserves further study.

## Conclusions

In summary, we have identified that a long noncoding RNA, *AFAP1-AS1*, is up-regulated in PDAC tissues and serves as a negative prognostic factor for lymph node metastasis, perineural invasion, and poor survival in PDAC patients. The value of *AFAP1-AS1* as a potential prognostic biomarker and/or therapeutic target in PDAC was supported by findings that knockdown of *AFAP1-AS1* in PDAC cells inhibits cell proliferation, motility, and invasiveness *in vitro*.

## Additional files

Additional file 1:
**The primers used for the qRT-PCR analysis.**
Additional file 2:
**Differentially expressed lncRNAs between PDAC tissues and CP tissues.**

## Abbreviations

PC: Pancreatic cancer
PDAC: Pancreatic ductal adenocarcinoma
CP: Chronic pancreatitis
lncRNA: Long noncoding RNA
AFAP1: Actin filament associated protein 1
*AFAP1-AS1*: Actin filament associated protein 1 antisense RNA
EMT: Epithelial Mesenchymal Transition
